# The Influence of Season on the Gonad Index and Biochemical Composition of the Sea Urchin *Paracentrotus lividus* from the Golf of Tunis

**DOI:** 10.1100/2012/815935

**Published:** 2012-05-03

**Authors:** Soumaya Arafa, Moncef Chouaibi, Saloua Sadok, Amor El Abed

**Affiliations:** ^1^Department of Valorisation and Conservation of food product, Ecole Supérieure des Industries Alimentaires de Tunis, 58 Avenue Alain Savary, Cité El Khadhra, Tunis 1003, Tunisia; ^2^Department of Biotechnology, Institut National des Sciences et Technologies de la Mer, 28 Rue 2 Mars 1934, Carthage, Salammbo, Tunis 2025, Tunisia

## Abstract

Seasonal variation in the gonad weight and biochemical composition of the sea urchin *Paracentrotus lividus* from the Golf of Tunis (Tunisia) were studied between September 2003 and August 2004. The highest gonad indices occurred in March (16.71%). The spawning period occurred between April and July and resulted in a fall in gonad indices to low level (7.12 ± 0.12%). Protein constituted the main component of the gonad, and lipid and carbohydrate were found at appreciable amounts. Consistent with the gonad cycle, sea urchin biochemical components showed clear seasonal variation with a significant decrease during the spawning period. The polyunsaturated fatty acid (PUFA) group was found at high level (40% of the total fatty acids). Of the PUFA group, eicosapentaenoic (C20:5 *n* − 3) and eicosatetraenoic (C20:4 *n* − 3) were the most abundant gonadal lipids. The level of PUFA was significantly affected by temperature variation showing an increase during the cold months and a decrease in the hot months.

## 1. Introduction

Sea urchin *Paracentrotus lividus* (Lamarck) is a widespread species in the Atlantic and the Mediterranean coasts and is subjected to intensive commercial fishing in several countries. This species was prised for its yellow gonads which have a caviar-like appearance and a bittersweet flavour [1]. Sea urchin gonads, also known as roe or uni, are a highly valued seafood commodity and are considered as delicacies in many parts of the world.

However, sea urchin gonad composition may exhibit wide variability, which can affect gonad quality. The factors that affect sea urchin gonad composition can be endogenous, exogenous, or both operating in concert. The endogenous factors are genetically controlled and are associated with the animal life cycle. Thus, as the gonads increase in size, somatic growth slows down and eventually stops. At this time, proteins and lipids are mobilised from the muscle and transferred to the gonads [2].

The exogenous factors, both environmental (temperature, salinity, etc.) and dietary (diet composition, feeding frequency, ration level, etc.), have also been reported to affect the proximate composition of the sea urchin gonads [3]. However, it is difficult to attribute the change in biological component to any specific environmental factor, such as food or temperature, because of their parallel variation.

Water temperature is one of the most important factors governing metabolic processes in sea urchins [4, 5]. Under conditions of unlimited food supply, an increase in temperature was found to induce an increase in food intake. Growth rate increases with temperature within certain species-specific ranges, but high temperatures result in negative instead of stimulatory effects. In a summary on ecology of the green sea urchin, Scheibling and Hatcher [6] concluded that feeding rates are not linearly related to temperature but generally show a strong relationship with the reproductive cycle which itself varies seasonally with temperature.

Lipids are important energy reserves that can store more energy per unit volume than proteins or carbohydrates [7]. In addition, lipids, such as phospholipids and cholesterol, are structural components of cell and subcellular membranes and vital for somatic growth [8–10]. Lipids are comprised of fatty acids (FA), some of which particularly dihomo-gamma linolenic (20:3 *n* − 6, DHGLA), arachidonic (20:4 *n* − 6, AA), eicosapentaenoic (20:5 *n* − 3, EPA), and docosahexaenoic (22:6 *n* − 3, DHA) are essential for a multitude of physiological functions in animals [11, 12].

The lipid composition of animals is not fixed. Diet and growth may exert strong influence on fatty acid profiles. The specificity of fatty acid synthesis and composition in different taxonomic groups is the basis for their wide use as biochemical markers of trophic and metabolic interactions in aquatic ecosystems [13].

Several studies have been undertaken on the biochemical composition of sea urchin gonads in different regions of the world in order to assess and improve nutrients that are important for these species [14, 15]. However, in Tunisia despite the economic importance of these species, such studies have not been conducted on the sea urchin *P. lividus*.

 In this study, the biochemical composition in relation to seasonal gonad indices variation was investigated.

## 2. Materials and Methods

### 2.1. Sample Collection

Sea urchins, *Paracentrotus lividus* (Lamarck), of commercial size (total wet weight 83.34 0.6 g) were collected from the intertidal zone of the Gulf of Tunis. Sea urchins (≈42 animals) were monthly collected between September 2004 and August 2005. To avoid spawning, the sea urchins were weighed and dissected immediately after collection in the field. The Gonads were removed, weighed, and stored in liquid nitrogen until analysis.

***Gonadosomatic Index* (%)**

The gonadosomatic index (GSI) of the sea urchin was calculated as a ratio of the gonad mass to the whole-body wet mass

(1) $$\text{Gonadosomatic}\;\text{index}=\frac{\text{Gonad}\;\text{weight}}{\text{Total}\;\text{weight}}\times 100.$$

### 2.2. Protein Analysis

Total protein was assayed using Sigma Kit 540 (Sigma chemical Co) as described. To ten *μ*L of sample tissue (homogenised in 1 mL ultra-pure water) was added 1.0 mL of Biuret reagent. Blank and standard samples were made using 10 *μ*L protein standards (80 mg mL^−1^ bovine serum albumin), respectively, added to 1 mL Biuret reagent for drowning a calibration curve. All test solutions were incubated at 20°C for 15 min. Absorbance of the incubated samples was read at 540 nm.

### 2.3. Carbohydrate Analysis

The carbohydrate content was analysed according to the method of Dubois et al. [16]. The sample (0.25 g) was homogenised in ultra-pure water (2 mL) and diluted to 20 *μ*g mL^−1^ with ultra-pure water. An aliquot, 50 *μ*L, of the homogenate was carefully pipetted into labelled Eppendorf tube, mixed with 50 *μ*L of 5% phenol and allowed to stand at 15°C for 20 min. To the resulting solution, 500 *μ*L of H_2_SO_4_ (98%) was added, cooled on ice, and subsequently centrifuged at 12000 g using a Sigma 3K30 centrifuge. After centrifugation, the supernatant was carefully transferred to a cuvette, and its absorbance was read at 492 and 620 nm using the spectrophotometer SLT spectra. Total carbohydrate absorbance was calculated using the following equation:

(2) $$\begin{array}{cc} & \text{Absorbance}\\ & \;\;={\text{Absorbance}}_{492\;\text{nm}}-\left({1.5\;\text{Absorbance}}_{620\;\text{nm}}-0.003\right). \end{array}$$

### 2.4. Lipid Extraction

Total lipid was extracted by the method of Folch et al. [17]. The samples (0.5 g) were homogenised in 10 mL of chloroform methanol (2 : 1, v/v) using a manual homogeniser. The samples were then centrifuged (1200 g, 5 mn) to separate the lipid containing organic layer from the aqueous layer. The organic layer was transferred in to a preweighed boiling tube and subsequently evaporated to dryness at 37°C under nitrogen. The dried tube containing lipid was cooled in a dessicator and reweighed. The weight of the crude lipid was calculated by subtracting the initial weight of the empty tube from the weight of the tube containing the dried lipid.

### 2.5. Analysis of Fatty Acid Composition

Fatty acid methyl esters (FAMEs) of lipids were prepared according to the method of Shirai et al. [18]. The total lipid was saponified with 0.5 mol L^−1^ NaOH in methanol for 15 min at 100°C. The recovered fatty acids were methylated with 14% boron trifluoride (BF_3_) in methanol for 20 min at 100°C. The fatty acid methyl esters were analysed by HP Gas Chromatograph (hp 5890) fitted with flame ionisation detector and an INNOWAX capillary column (30 m × 0.25 mm × 0.25 *μ*m,). Helium was used as a carrier gas. The oven temperature was programmed to rise from 160 to 240°C at a rate of 4°C min^−1^. The injector temperature was programmed to rise from 100 to 250°C at a rate of 30°C/min. The detector temperature was 250°C. FAME peaks of the samples were identified by comparison with retention times of known fatty acid methyl ester standard mixture supplied by Supleco and fatty acid methyl esters standard prepared from menhaden oil. Varian Star integration package was used to integrated and calculated peak areas.

## 3. Statistical Analysis

The data are expressed as means and standard deviations (±). Homogeneity of the data was explored with the Levene test. The arcsine transformation method was applied to transform data that were not normally distributed. Data with homogeneous variances were analyzed using Analysis of Variance (ANOVA), and the Tukey's multiple comparisons to determine differences among independent factors. All statistical analyses were performed with the use of the software package SPSS for windows, version 8.

## 4. Results

### 4.1. Gonad Index

**Figure 1:**
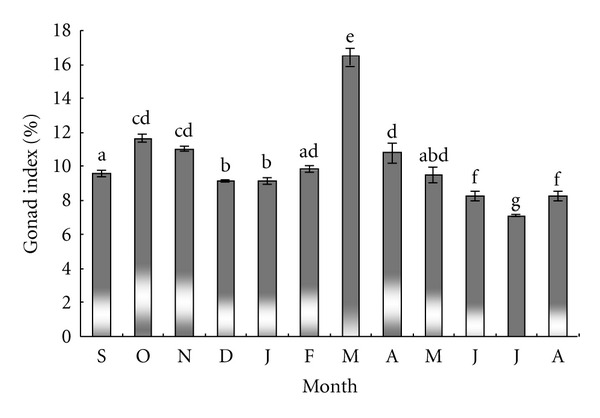
*Paracentrotus lividus*. Monthly variation of gonad index. Columns with the same letter are not significantly different.

The monthly changes in gonad mass of sea urchin are summarized in Figure 1. Sea urchin *P. Lividus* gonadal index reached a maximum value of 16.46 ± 2.59% in March, and then decreased steadily to a minimum value of 7.12 ± 0.12% in July (*P* < 0.01). The index increased progressively between August (8.25 ± 0.35%) and November (11.03 ± 0.24%**)** (*P* < 0.05), then declined in December (9.13 ± 0.12%) to remain constant until February.

### 4.2. Protein Content

**Figure 2:**
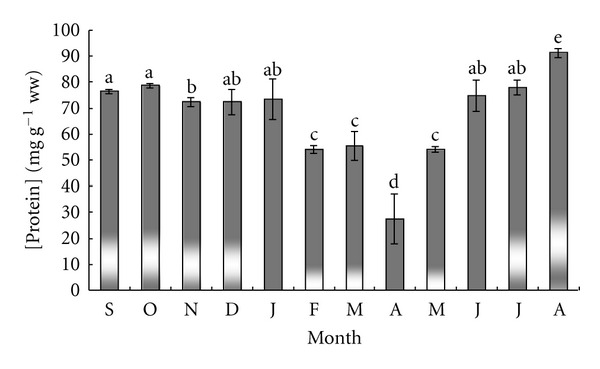
Monthly variation of protein levels in sea urchin gonads. Columns with the same letter are not significantly different.

Figure 2 presents the monthly protein content of sea urchin gonads. Protein level remained relatively constant between September and January (74.79 ± 1.10 mg g^−1^ wet weight), and decreased to a minimum value of 27.46 ± 2.51 mg g^−1^ wet weight in April (*P* < 0.05). From the lowest level unregistered in April, protein content increased progressively to a maximum value (91.26 ± 1.02 mg g^−1^ wet weight) in August (*P* < 0.05).

### 4.3. Carbohydrate Content

**Figure 3:**
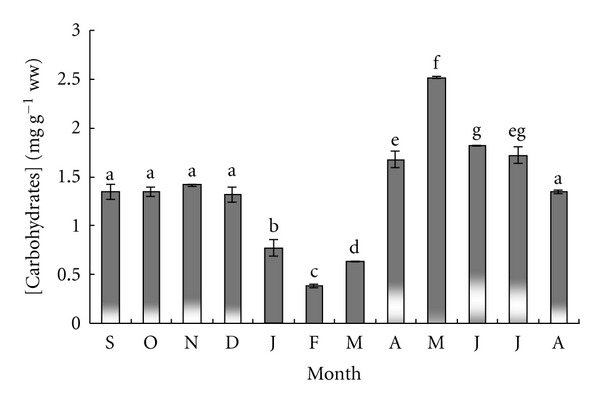
Monthly variation of carbohydrate levels in sea urchin gonads. Columns with the same letter are not significantly different.

Figure 3 shows that carbohydrate contents remained constant (1.34 ± 0.05 mg g^−1^ wet weight) between September and December and declined in January (0.09 mg g^−1^ wet weight, *P* < 0.05) and February (0.38 ± 0.02 mg g^−1^ wet weight, *P* < 0.05). During the spring and winter seasons, the gonad carbohydrate content increased steeply and reached a maximum level in March (2.51 ± 0.02 mg g^−1^ wet weight, *P* < 0.05). In summer, the gonad carbohydrate content decreased again and reached a value of 1.34 ± 0.02 mg g^−1^ wet weight in August.

### 4.4. Lipid Content

**Figure 4:**
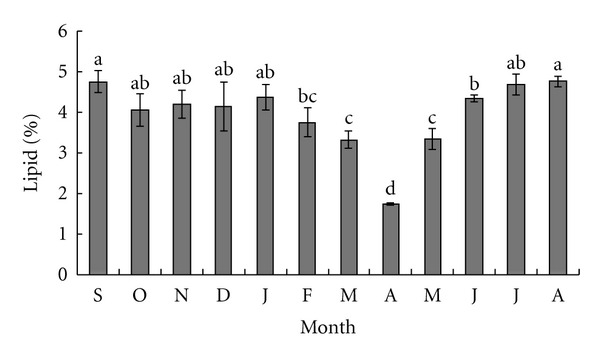
Monthly variation of lipid levels in sea urchin gonads. Columns with the same letter are not significantly different.

Figure 4 shows lipid content monthly variations in sea urchin gonads. In contrast to proteins and carbohydrates, lipids showed relatively constant level throughout most season (4.3 ± 0.08%), except spring season with the lowest value unregistered in April (1.73 ± 0.02%, *P* < 0.05).

### 4.5. FA Content

Fatty acid profiles of sea urchin gonads are shown in Table 1. In this study, only the monthly variation of gonad fatty acids content that contributed more than 2% of the total fatty acids is included in the discussion.

PUFA represented the highest proportions, contributing to more than 40% of the total gonad fatty acids. SFAs which were the second dominating group, represented 25.6% of the total gonad fatty acids; the MUFA group represented 14.25% of total fatty acids.

The highest levels of the PUFA were found in the winter and in the spring, while the lowest occurred in the summer (*P* < 0.01). Eicosapentaenoic (C20:5 *n* − 3, EPA), docosadienoic (C20:2), and docosatetraenoic (C20:4 *n* − 3) were the major PUFA in the sea urchin gonads. EPA, which was the major compound, comprised more than 40% of the total PUFA.

The seasonal variations of PUFA were primarily due to changes in C20:5 *n* − 3 and C20:4 *n* − 3. The fatty acid C20:2, which was proportionally the second important PUFA, did not show significant seasonal variations.

The trend of changes in PUFA and SFA contents was totally opposite, with a significant reduction of the gonad SFA content during the period ranging between November and March. The main saturated fatty acids were C16:0, C14:0, and C18:0. The pattern of changes of C16:0 and C14:0 was similar throughout the year. In the winter, the level of the C16:0 and the C14:0 fatty acids decreased to a minimum of 10.76% and 4.52%, respectively. Similarly, in the summer, those fatty acids increased progressively to a maximum percentage (17.03% and 8.17% resp.) in August. The level of C18:0 remained stable throughout the year with a mean value of 2.78%.

The MUFA content was lower in the autumn and in the beginning of the summer with minimum value of 11.75% in December. Higher values of MUFA were evident in July (16.73 ± 0.78%), August (16.52 ± 1.02%), and September (17.63 ± 052%). The dominant MUFAs were C18:1 *n* − 9 and the C18:1 *n* − 7. In the winter (December), the percentage of the C18:1 *n* − 9 decreased to the lowest value of 3.67% and then increased progressively to the highest level of 11.49% in August. During the same period, the percentage of C18:1 *n* − 7 dropped down to a minimum of 0.31%, with a percentage significantly lower (*P* < 0.01) than that of the C18:1 *n* − 9. Between October and January, the C18:1 *n* − 7 percentages remained significantly higher than that of the C18:1 *n* − 9.

### 4.6. Variation of C18:1 *n* − 9/C18:1 *n* − 7 Ratios

In October, the ratio of C18:1 *n* − 9/C18:1 *n* − 7 was 0.56 and remained around that value through November and December. In January, it increased significantly (*P* < 0.05; 0.66) to reach a maximum value of 36.67 in August (Table 1).

## 5. Discussion

Various studies have investigated different aspects of the gonad cycle in the sea urchin, *Paracentrotus lividus* [19–21]. In Mediterranean population, single annual spawning period [22] and two annual spawning periods [14, 23] have been reported. Previous studies revealed that, within the same area (Ireland), both single and double spawning may occur [24]. The current study confirmed the presence of a single annual spawning of the sea urchin *P. lividus* from the Gulf of Tunis (occurring in spring) with a minimum gonad index value in the summer. A similarity in the reproductive pattern of echinoids from temperate areas, with a nutrient storage period in autumn and winter and a long spawning period during summer, was equally reported [20]. However, Spirlet et al. [21] revealed a significantly (*P* < 0.05) lower value of GSI (ranging between 6% and 12%) of *P. lividus* in natural condition. The difference in gonad index was probably due to sea urchin provenance, which in our study came from intertidal zone with abundant food, and, therefore, high GI values were expected. [25] and Spirlet et al. [21] reported similar pattern of gonadal index variation. They also revealed that the gonadal growth of *P. lividus* occurs during the coldest months of the year and during the period of shortest days, suggesting that both parameters are responsible for gonadal growth. In addition, Regis [23] and Byrne [25] demonstrated that a decrease in temperature during autumn is an important factor for initiating gonad growth in Mediterranean and Irish populations of *P. lividus*. However, an increased temperature in spring acted as a catalyst for gametogenic processes, and thereafter spawning [20].

Gross biochemical composition of Echinoidea was generally independent of species and geographical location [26]. The biochemical composition of *P. lividus* gonad from Tunisia is comparable to that observed in sea urchins from other parts of the world [27, 28], with important reserves of protein, relatively abundant quantities of lipids and lower levels of carbohydrate. However, in echinoids, such biochemical composition varies seasonally [14, 26, 29] and was related to food quality and availability [15, 30], to temperature variation [21] and to reproductive cycle [14, 31]

Our results revealed that proteins were found to be the main component of *P. lividus* gonads [15, 27]. The protein content was related to the gonad's reproductive cycle showing important levels prior to spawning. Fernandez [27] reported similar results. An inverse relationship between protein reserve and gametogenesis was equally found in [32]. In general, energy is stored prior to gametogenesis when food is abundant and utilised subsequently in the production of gametes at high metabolic demand [25].

The carbohydrate was identified as a primary source of energy for gonad growth and gametogenesis in sea urchin [15, 33]. Thus, carbohydrate reached a minimum level when gonad mass increased to a maximum value. Similarly, Patrick et al. [34] revealed this inverse relationship between gonad mass and carbohydrate levels in oyster (*Crassostrea gigas*). These authors reported that sexual maturation in oyster was closely associated with the carbohydrates breakdown independently of the rearing site. Moreover, carbohydrates are used to produce energy to support the temperature decrease during the winter [35]. In this study, the increase of carbohydrate level during the spring season is probably due to food abundance.

### 5.1. Lipid and Fatty Acids Composition of the Gonads

Results showed that gonad total lipids in the *P. lividus* were similar to those of other echinoids [36–38]. Seasonal variation in the total lipids was less evident than for the other biochemical components.

Fatty acids profiles were also identified during this study. The polyunsaturated fatty acids constitute the highest proportion of the total fatty acids identified in the gonads (Figure 1). This finding is consistent with published data [10, 37, 39]. Numerous researchers have reported that ovaries contain large amounts of EPA and DHA [40, 41]. In this study, EPA was observed to be the major polyunsaturated fatty acids in sea urchin gonad. In contrast, the level of DHA, which was thought to be one of the major fatty acids in marine organisms, was only between 0.47 and 2.2% of the total fatty acids. Similarly, Pathirana et al. [37] found 1–2.5% DHA and a high level of EPA in the gonads of *S. droebachiensis*. Mai et al. [42] reported that the high proportion of EPA is a reflection of the presence of a macroalgal material in the diet of sea urchin, since this fatty acid is found in macroalgal species such as *Laminaria digitata* and *Alaria esculenta*. In this study, the C20:2, the C20:3 and the C20:4 were present in important percentages. These fatty acids were found to be the major contributors in sea urchin tissues, even when they were not detected in the diet [37]. It appears that these fatty acids are synthesized by sea urchins from lower fatty acid precursors [43, 44]. These fatty acids are also known to have structural functions [8].

The major saturated fatty acids were C16:0, C14:0, and C18:0 with dominance of C16:0 (>60% of the SFA). This is in accordance with the available published data on fatty acid composition of wild sea urchins [8, 37, 45].

Furthermore, the present study showed that fatty acids C18:1 *n* − 9 and C18:1 *n* − 7 were the dominant MUFA. In other studies, C20:1 was, however, the dominant fatty acids in sea urchin gonad [8, 37]. The presence of the fatty acid C20:1 has not been commonly reported as being typical of marine lipids. Ackman and Hooper [46] stated that in marine animals, such as periwinkle (*Littorina littorea*), moon snail (*Lunata triseriata*), and sand shrimp (*Crangon septem-spinosus*), the C20:1 did not exceed 0.2% of the total fatty acid content. The formation of the C20:1 in the sea urchin gonadal lipids may be biosynthetic in origin since it was not reported in seaweeds, which is the natural diet of sea urchins [47].

### 5.2. Monthly Variation of the Sea Urchin Gonad Fatty Acid Composition

In this study, the influence of the environmental conditions (food type and temperature variation) on FA composition should be discussed with regards to the reproductive cycles which can also contribute to the variation of FA composition in sea urchin gonads.

During this study, a seasonal change of PUFA in sea urchin gonads was also recorded. Naumenko and Kostetsky [48] reported similar results, showing an inverse PUFA level in marine invertebrates during the winter and spring. This is further supported by the fact that changes in saturation of fatty acids depend on temperature, and several authors consider them as adaptive reactions to sustain membrane fluidity [12, 18, 49, 50]. For instance, Farkas and Herodek [51, 52] and Farkas [53] revealed an increased amount of C20 and C22 PUFAs in some crustacean plankton, in response to environmental temperature decrease.

In addition, animals are known to receive considerable amounts of lipids via their diet [18, 38] which type was found to alter the fatty acid composition of certain herbivorous copepods such as *Calanus spp* [54] or the sea urchin *P. miliaris* [43]. For example, PUFA, namely, the C20:5 and the C20:4 fatty acids, fluctuated to maximum levels in spring and to minimum levels in summer and in winter, since C20:5 and C20:4 fatty acids were found in large quantities in macroalgal species such as *Porphyridium*,* Ascophyllum*, and *Laminaria* [55] which were abundant in spring. However, DHA, which was associated with summer-carnivorous feeding, picked in August [56, 57].

 The ratio of C18:1 *n* − 9/C18:1 *n* − 7 fatty acids in the gonads of *P. lividus* also showed a wide range in values, particularly between sea urchins collected at different seasons. The gonads of sea urchins, collected in summer and spring, exhibited the highest ratio of C18:1 *n* − 9/C18:1 *n* − 7 fatty acids. Such ratio has been found to be good indication of the contribution of bacteria to the nutrition of marine organisms [58]. In addition, the proportions of odd-chain and branched fatty acids within the lipids fraction has also been used to indicate the level of dietary bacterial input [59]. It is well established that sediment contains a high level of odd-chain and branched fatty acids, which are believed to be of bacterial origin [59–61]. Our results suggested that in summer and spring sea urchins may acquire some of their fatty acids from the substratum, since algal foods were rare. It also suggested that sea urchins have a high bacterial and/or diatom input in their diet at these seasons. Cook et al. [43] mentioned that sea urchin *P. miliaris* showed a wide range of C18:1 *n* − 9/C18:1 *n* − 7 ratio, which varied with the location from where sea urchins were collected. However, to our knowledge, no study was initiated to examine such seasonal variation, and further investigations are needed.

## Figures and Tables

**Table 1:** *Paracentrotus lividus*. Variation of fatty acid composition of sea urchin gonad during the months of the year (from September 2003 to August 2004). Values are means (*n* = 6, ±SD) of gonad samples pooled from 40 sea urchins in each case. Different letters (a, b, c, and d) indicate significant differences for data in the same column.

Fatty acid	September	October	November	December	January	February	March	April	May	June	July	August
C14:0	6.84 ± 1.50^ac^	6.45 ± 0.4^a^	5.93 ± 0.18^a^	6.13 ± 0.24^a^	5.83 ± 0.29^a^	4.53 ± 0.54^b^	5.87 ± 1.56^a^	5.47 ± 0.12^ab^	6.06 ± 0.59^a^	7.08 ± 1.5^c^	7.08 ± 1.5^c^	8.17 ± 0.5^d^
C15:0	0.70 ± 0.07^a^	1.12 ± 0.07^a^	1.01 ± 0.1^a^	0.96 ± 0.01^a^	1.00 ± 0.05^a^	0.80 ± 0.07^a^	0.70 ± 0.07^a^	0.53 ± 0.27^a^	0.69 ± 0.00^a^	0.65 ± 0.07^a^	0.65 ± 0.06^a^	0.70 ± 0.00^a^
C16:0	17.52 ±.18^a^	17.29 ± 0.36^a^	15.37 ± 1.33^b^	14.48 ± 3.42^c^	10.77 ± 4.67^d^	12.76 ± 0.7^e^	14.10 ± 2.12^c^	13.68 ± 0.44^c^	14.50 ± 0.81^bc^	15.18 ± 2.18^b^	16.11 ± 0.56^b^	17.30 ± 0.16^a^
C17:0	0.69 ± 0.16^a^	0.45 ± 0.01^a^	0.37 ± 0.06^a^	0.35 ± 0.01^a^	0.40 ± 0.2^a^	0.54 ± 0.76^a^	0.76 ± 0.29^ab^	1.31 ± 0.11^b^	0.36 ± 0.23^a^	1.41 ± 0.16^b^	1.25 ± 0.2^b^	0.73 ± 0.01^ab^
C18:0	1.84 ± 0.19^a^	3.02 ± 0.2^b^	2.83 ± 0.63^b^	2.43 ± 0.25^ab^	2.34 ± 0.29^ab^	1.98 ± 0.15^a^	2.16 ± 0.25^a^	2.05 ± 0.07^a^	2.08 ± 0.06^a^	2.25 ± 0.19^a^	2.11 ± 0.18^a^	1.84 ± 0.08^a^
C19:0	—	—	—	—	—	—	—	—	—	—	—	—
C20:0	0.40 ± 0.01^a^	0.38 ± 0.02^a^	0.45 ± 0.04^a^	0.44 ± 0.00^a^	0.22 ± 0.23^a^	0.39 ± 0.03^a^	0.39 ± 0.04^a^	0.35 ± 0.02^a^	0.34 ± 0.04^a^	0.37 ± 0.01^a^	0.37 ± 0.01^a^	0.40 ± 0.01^a^
C22:0	—	—	—	—	—	—	—	—	—	—	—	—
*∑* SFA	27.99 ± 0.45^a^	28.72 ± 0.63^ad^	25.96 ± 0.13^b^	24.79 ± 1.36^b^	22.56 ± 0.52^c^	21.00 ± 2.36^c^	21.07 ± 0.41^c^	23.38 ± 1.36^b^	24.04 ± 0.25^b^	26.94 ±2.47^ab^	27.57 ± 2.36^a^	29.14 ± 1.69^d^
C14:1	0.25 ± 0.07^a^	0.19 ± 0.09^a^	0.12 ± 0.07^a^	0.22 ± 0.23^a^	0.20 ± 0.12^a^	0.19 ± 0.02^a^	0.44 ± 1.16^a^	0.35 ± 0.03^a^	0.28 ± 0.03^a^	0.51 ± 0.07^a^	0.40 ± 0.00^a^	0.25 ± 0.00^a^
C16:1 *n* − 7	2.23 ± 0.05^a^	0.44 ± 0.05^b^	1.08 ± 0.83^bc^	0.29 ± 0.02^b^	1.25 ± 0.78^c^	2.30 ± 0.52^a^	1.50 ± 1.7^c^	1.33 ± 0.93^c^	0.43 ± 0.13^b^	0.56 ± 0.05^b^	1.76 ± 0.51^ac^	2.68 ± 0.12^a^
C18:1 *n* = 9	6.85 ± 0.84^ae ^	3.19 ± 0.05^b^	4.14 ± 1.41^bd^	2.39 ± 0.36^c^	2.83 ± 0.28^bc^	3.01 ± 0.88^b^	4.56 ± 0.74^d^	5.93 ± 0.17^ae^	6.62 ± 0.27^ae^	7.01 ± 0.84^e^	10.42 ± 4.13^f^	11.50 ± 2.13^f^
C18:1 *n* = 7	6.51 ± 0.19^a^	6.27 ± 0.12^a^	5.56 ± 1.3^a^	5.77 ± 0.23^a^	7.28 ± 0.7^b^	3.68 ± 0.12^cd^	4.15 ± 0.51^c^	3.33 ± 0.22^cd^	3.12 ± 0.27^cd^	2.88 ± 0.19^d^	1.14 ± 0.61^e^	0.31 ± 0.01^e^
C20:1 *n* − 9	0.00^a^	1.04 ± 0.02^b^	0.99 ± 0.27^b^	1.04 ± 0.12^b^	0.83 ± 0.07^b^	0.77 ± 0.1^b^	1.75 ± 0.3^b^	1.36 ± 0.05^b^	1.11 ± 0.1^b^	1.13 ± 0.27^b^	1.13 ± 0.27^b^	0.00^a^
C22:1 *n* − 11	1.78 ± 0.02^a^	1.66 ± 0.05^a^	1.99 ± 0.12^a^	2.04 ± 0.13^a^	1.44 ± 0.04^a^	2.47 ± 0.14^a^	1.79 ± 0.2^a^	1.72 ± 0.1^a^	1.89 ± 0.42^a^	1.89 ± 0.11^a^	1.88 ± 0.13^a^	1.78 ± 0.03^a^
*∑* MUFA	17.63 ± 0.52^a^	12.79 ± 0.76^bc^	13.88 ± 0.83^bd^	11.76 ± 2.10^c^	13.83 ± 0.72^bd^	12.42 ± 1.36^b^	14.19 ± 0.42^d^	14.02 ± 0.85^d^	13.45 ± 0.1^bd^	13.98 ± 0.34^d^	16.73 ± 0.78^a^	16.52 ± 1.02^a^
C18:2 *n* − 6	0.34 ± 0.18^a^	1.51 ± 0.26^b^	3.14 ± 0.37^c^	1.93 ± 0.15^b^	2.31 ± 0.15^b^	2.09 ± 0.22^b^	1.69 ± 0.2^b^	1.94 ± 0.07^b^	2.30 ± 0.35^b^	2.26 ± 0.18^b^	0.85 ± 0.33^ab^	0.34 ± 0.03^a^
C18:3 *n* − 3	2.00 ± 0.83^a^	1.86 ± 0.19^a^	1.49 ± 0.46^a^	2.86 ± 0.96^ab^	3.37 ± 0.27^b^	4.25 ± 0.6^c^	2.28 ± 0.36^a^	2.81 ± 0.25	2.81 ± 0.01^ab^	1.82 ± 0.23^a^	1.82 ± 0.03^a^	2.00 ± 0.23^a^
C20:2	11.26 ± 0.21^a^	10.06 ± 1.37^b^	12.22 ± 0.97^c^	12.27 ± 2.01^c^	9.24 ± 0.21^b^	9.22 ± 0.25^be^	8.59 ± 1.11^c^	7.85 ± 0.11^d^	8.77 ± 1.13^ce^	9.43 ± 0.21^b^	11.48 ± 3.18^a^	11.26 ± 1.25^a^
C20:3	2.68 ± 0.41^ab ^	2.58 ± 0.4^a^	3.47 ± 0.55^b^	3.47 ± 0.35^b^	2.83 ± 0.12^ab^	2.70 ± 0.31^ab^	2.91 ± 0.39^ab^	2.75 ± 0.15^ab^	2.61 ± 0.29^a^	2.59 ± 0.41^a^	3.43 ± 0.25^b^	3.90 ± 0.25^b^
C20:4 *n* − 3	7.91 ± 0.69^a^	8.22 ± 0.37^a^	8.37 ± 0.45^ab^	8.02 ± 1.00^ab^	8.62 ± 0.4^ab^	7.73 ± 0.41^a^	7.66 ± 1.09^a^	8.19 ± 0.23^ab^	8.05 ± 0.49^ab^	7.47 ± 0.69^a^	6.01 ± 0.68^c^	5.69 ± 0.65^c^
EPA C20:5 *n* − 3	16.80 ± 1.48^a^	16.05 ± 1.45^ab^	15.30 ± 1.69^b^	15.46 ± 2.36^b^	17.89 ± 0.54^c^	19.39 ± 0.85^d^	20.93 ± 4.28^e^	21.05 ± 0.1^e^	16.70 ± 0.93^a^	16.17 ± 1.48^a^	14.90 ± 0.44^bf^	14.59 ± 0.36^f^
DHA C22:6 *n* − 3	1.01 ± 0.05^ab^	0.78 ± 0.1^a^	1.14 ± 0.24^ab^	1.28 ± 0.23^ab^	0.90 ± 0.09^a^	0.57 ± 0.09	1.60 ± 0.2^b^	0.47 ± 0.2^a^	0.86 ± 0.08^a^	1.00 ± 0.07^ab^	1.93 ± 0.15^b^	2.21 ± 0.25^b^
*∑* PUFA	42.09 ± 0.54^a^	41.06 ± 0.63^ac^	45.13 ± 1.02^b^	45.30 ± 1.56^b^	44.99 ± 0.92^b^	45.96 ± 0.56^b^	45.66 ± 2.02^b^	45.06 ± 0.63^b^	42.1 ± 0.7^a^	40.74 ± 1.02^cd^	40.42 ± 2.13^cd^	39.99 ± 0.22^d^
C18:1 *n* = 9/ C18:1 *n* = 7	1.05	0.5	0.74	0.41	0.38	1.22	1.09	1.78	2.12	2.43	9.17	36.67
